# Anterior cruciate ligament injury and its postoperative outcomes are not associated with polymorphism in *COL1A1* rs1107946 (G/T): a case–control study in the Middle East elite athletes

**DOI:** 10.1186/s13018-022-03341-9

**Published:** 2022-10-21

**Authors:** Seyed Peyman Mirghaderi, Maryam Salimi, Majid Kheirollahi, Seyed Mohammad Javad Mortazavi, Hossein Akbari-Aghdam

**Affiliations:** 1 Joint Reconstruction Research Center (JRRC), Tehran University of Medical Sciences, Tehran, Iran; 2 Students’ Scientific Research Center (SSRC), Tehran University of Medical Sciences, Tehran, Iran; 3 Molecular Biology and Medical Genetics, School of Medicine, Isfahan University of Medical Sciences, Isfahan, Iran; 4 Department of Orthopedic Surgery, School of Medicine, Isfahan University of Medical Sciences, Isfahan, Iran

**Keywords:** Anterior cruciate ligament, Collagen, Genetic variation, Patient-reported outcome measures, Single-nucleotide polymorphisms

## Abstract

**Background:**

It is unclear what role *COL1A1* polymorphisms play in anterior cruciate ligament **(**ACL) injury pathophysiology. The present study investigated the relationship between *COL1A1*-1997 guanine (G)/thymine (T) (rs1107946) polymorphism and ACL injury. Moreover, the possible effect of this polymorphism on the postoperative outcomes of ACL reconstruction surgery was evaluated.

**Methods:**

This prospective case–control study was performed on 200 young professional men with an ACL tear who underwent arthroscopic ACL reconstruction surgery. Moreover, 200 healthy athletes without a history of tendon or ligament injury who were matched with the case group were selected as the control group. DNA was extracted from the leukocytes of participants, and the desired allele was genotyped. Clinical outcomes were collected for the case group before and one year after surgery.

**Results:**

The genotype distribution was in accordance with the Hardy–Weinberg principle. In the ACL injury group, the G allele frequency was non-significantly higher than the healthy controls, with an odds ratio [95% CI] of 1.08 [0.79–1.47] (*P* = 64). We did not find a significant difference between the genotype of individuals—GG, GT, and TT—in the case and control groups (*P* > 0.05). Clinical outcomes of the ACL tear group were significantly improved in terms of preoperative values. However, none of them were significantly different between the three genotypes (GG, GT, and TT).

**Conclusion:**

According to the findings of the present investigation, single-nucleotide polymorphism (SNP) at *COL1A1* rs1107946 (G/T) was not a predisposing genetic factor for ACL injury in a young professional male athlete population in the Middle East. Furthermore, patients' responses to treatment were not different between distinct genotypes.

*Level of evidence* III.

## Highlights

- Collagen gene variations seemed to have an association with anterior cruciate ligament (ACL) injury among professional athletes.
- This study shows no association of a type of COL1A1 polymorphism ACL injury in a young professional male athlete population.
- Different genotypes of COL1A1 have similar outcomes in ACL reconstruction surgery.

## Introduction

Anterior cruciate ligament (ACL) injury is one of the most commonly reported sports injuries with a high cost and disastrous burden [1, 2]. The ACL injury is prevalent and may occur with concomitant meniscal injury, medial collateral ligament (MCL) tear, and knee cartilage damage [3–7]. The players of specific sports, such as basketball, soccer, and football, are at a higher risk of ACL injury, which can potentially cause severe disability for athletes [8, 9].

Although various underlying risk factors have been known to contribute to ACL tear, the detailed pathophysiology of the problem is still vague [10, 11]. Understanding the etiology, mechanism, and risk factors of ACL injury could improve the prevention of ACL injuries and would be useful in anticipating the prognosis of patients. Biological mechanisms underlying non-contact soft tissue injuries in musculoskeletal systems are largely unknown. There is evidence that genetic factors are associated with susceptibility to sport injuries and may play a significant role in the recovery time [12–14]. Several studies have confirmed the role of familial and genetic predisposing factors in increasing the risk of ACL injury [1]. Genetic variants in type I collagen as a major constituent of ligament tissue matrix have been investigated [1, 15, 16].

Type I collagen is the primary fibrillar collagen found in bones, tendons, and ligaments. This heterotrimer molecule consists of two α_1_ chains and one α_2_ chain encoded by *COL1A1* (chr17q21.33) and *COL1A2* (chr7q21.3) genes, respectively [16, 17]. Similar to many medical conditions in which familial genetic linkage has been found, researchers are interested in collagen gene variations in ACL injured patients to find a strong genetic predisposition and valid prognostic tool [15, 16, 18–24]. Although various polymorphisms in different loci of collagen genes have been tested in distinct ethnicities, there is still much to investigate. Two single-nucleotide polymorphisms (SNPs) in *COL1A1* have gained the most attention, namely *COL1A1* rs1800012 (G/T) and rs1107946 (G/T) [15, 18, 19]. The rs1800012 is an SP1-binding site within intron 1 at nucleotide 1023 and initially was shown to be associated with the risk of ACL injury [15, 25, 26]. The second polymorphism has been identified in the proximal promoter of *COL1A1*, at position − 1997 relative to the transcription start site (rs1107946). This polymorphism is related to bone mineral density [27, 28], distal radius fracture [29], muscle injury [30], and keloid scar formation at the end of the wound healing process [31].

However, little is known about the *COL1A1* rs1107946 polymorphism in the ACL injury. Despite extensive efforts, the results of the previous studies on these two sites did not reach a consensus [15]. An essential but neglected factor is that genetics studies in a population are highly variable and should be interpreted cautiously. Many studies confirmed the role of *COL1A1* rs1107946 polymorphism in musculoskeletal soft tissue injury among athletes [19, 23], while some investigations did not support this idea [18, 20, 24].

Furthermore, the clinical applications of these genetic evaluations toward a better future medical practice need to be addressed. Genetic studies have shown that future therapeutic approaches may get toward more specific and individualized agents or targeted therapy to obtain maximum efficacy and lower complications [32]. The ACL injury, in most cases, requires surgery, and the postoperative outcome has been the subject of numerous investigations and uncertainty. However, the patient-reported outcome measures (PROMs) are gaining much greater attention in choosing the appropriate management [33].

We hypothesized that *COL1A1* rs1107946 (G/T) polymorphism is a risk factor for ACL injury in a population of elite sports players. Thus, in this study, we compared the SNP at the *COL1A1* rs1107946 (G/T) site in a population of athletic Middle Eastern young men with ACL injuries with a matched control group. Furthermore, after a year of follow-up, we evaluated whether this SNP could be related to the postoperative outcomes of ACL reconstruction surgery.

## Materials and methods

### Study design and participants

After receiving the confirmation of an institutional review board, this prospective case–control study (Level of evidence = III) was conducted from 2017 to 2021 at our tertiary care center: Kashani hospital, Isfahan, Iran (Ethic code: IR.MUI.REC.1396.1.002). The inclusion criteria entailed: 1) Professional athlete for ≥ 2 years, 2) Affected by isolated ACL injury diagnosed with both physical examination and magnetic resonance imaging (MRI) of the knee, 3) Injured in sports activities during the last month, 4) Older than 18 years, 5) Participating in the sports of soccer, volleyball, basketball, and handball, and 6) Otherwise medically healthy and not consuming any medications or not being diagnosed with any other disease. Patients with a history of other significant comorbidities in the knee (e.g., other ligaments or tendon tear, knee degenerative joint disease, meniscus tear, or bone fractures), previous knee surgery, connective tissue disease (i.e., Marfan, Ehlers-Danlos syndrome or autoimmune disorders) were excluded. All of the included patients were provided information about the investigation, and written consent was received from them.

**Fig. 1:**
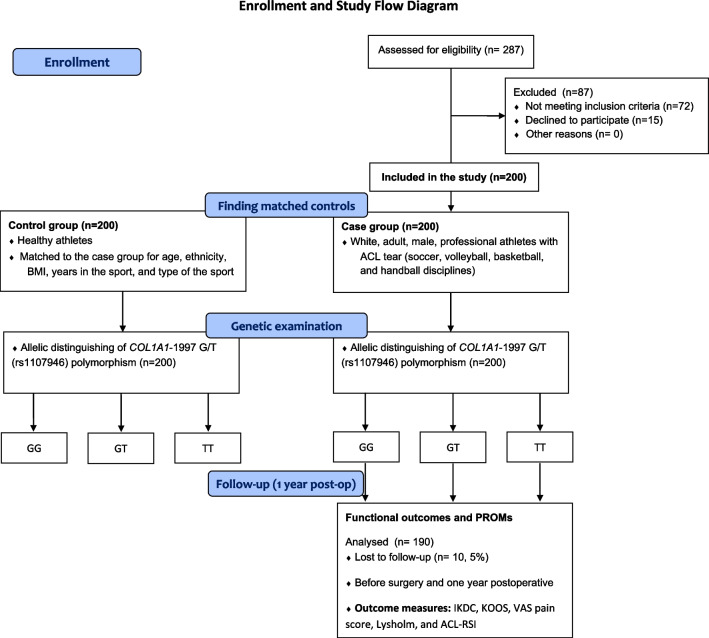
Patients’ enrollment process and study flow diagram

After screening a total of 287 white male professional athletes with an ACL tear, 200 were selected for the current study. They were referred to our center of excellence for orthopedic surgery and underwent arthroscopic ACL reconstruction surgery with the allograft augmentation of the hamstring by one experienced knee surgeon (H.AA). For controls, 200 healthy athletes without a history of tendon or ligament injury were selected contiguously from our national sports leagues and were matched to the case group for age, ethnicity, body mass index (BMI), years in the sport, and type of the sport (Fig. 1). The same ten-week physical therapy program was provided in a rehabilitative center to all patients postoperatively.

### DNA extraction and genotyping

Based on the recommendations of genotype–phenotype association studies [34, 35], we performed genomic DNA extraction from the leukocytes of the peripheral blood samples. To this end, 5 ml blood specimens were obtained from the antecubital vein and were preserved at − 20 °C until DNA extraction. DNA extraction was carried out following the protocol of previous studies [21]. The allelic distinguishing of *COL1A1*-1997 G/T (rs1107946) polymorphism was performed by TaqMan Pre-Designed SNP Genotyping Assays (Assay ID: C___7477171_10, Applied Biosystems, USA). This includes primers and fluorescently labeled probes (FAM and VIC) for allele detection. Amplification was completed by real-time polymerase chain reaction (PCR) (StepOne, Applied Biosystems, USA). During the thermal cycler, an initial step is carried out at 95 °C for 5 min, then 45 cycles of denaturation at 94 °C for 15 s, followed by annealing at 60 °C for 1 min [18]. For quality control, negative and positive controls were utilized during the polymerase chain reaction (PCR) process to detect polymorphism. Two blinded independent investigators numerated the genotyping results and provided them for further clinical investigations [18].

### Follow-up and functional outcome measures

The ACL-reconstructed patients underwent precise physical examination and subjective evaluations before surgery and one year postoperative. The anterior drawer tests (ADT) and Lachman tests were performed as explained by an experienced knee surgeon (H.AA) [36]. The injured knee was compared to the opposite side in terms of forwarding displacement, and the millimeter difference was documented as the degree of ACL laxity. The values (mm) were measured clinically with a ruler and compared to the normal site by our senior knee surgeon during the physical examination.

The collected PROMs encompassed International Knee Documentation Committee (IKDC) questionnaire [37], Knee Injury and Osteoarthritis Outcome Score (KOOS) [38], Visual Analog Scale (VAS) pain score [39, 40] in the knee region, Tegner Lysholm Knee Scoring Scale [41], and ACL-Return to Sport after Injury (ACL-RSI) scale.

The IKDC is an 18-item subjective scale, region-specific questionnaire for measuring symptoms, daily function, and sports activity in patients with knee disorders [37]. The KOOS tool assessed five distinct functional outcomes of the knee, including pain, stiffness and other symptoms, activities of daily living (ADLs), sport and recreation, as well as the quality of life. Each subscale is scored separately from 0 to 100 [38]. Tegner Lysholm knee score is another evaluation scale for the ADLs of the patients with knee disorders containing eight factors contributing to a maximum score of 100 [41]. Tegner Lysholm's tool includes items for support, limp, squatting, pain, instability, swelling, locking, and stair climbing. The ACL-RSI is a 12-item scale that evaluates psychological preparedness to return to sport. The ACL-RSI addresses three psychological factors, namely confidence in knee work, emotions, and risk assessment. The higher score (range: 1–10) represents the greater preparedness for returning to sport [42].

### Statistical analysis

We reported the descriptive statistics of quantitative data as mean ± SD and qualitative data as the frequency in the tables. The Chi-square or Fisher’s exact test was applied to assess the allelic difference between cases and controls. We compared genotype between the participants in the case and control groups in three ways because we did not have insight into the mode of inheritance for the minor allele: 1) inheritance was considered codominant, and an association test was performed in the 2 (phenotype) × 3 (genotype) table, 2) the minor allele inheritance was considered dominant, and the homozygote and heterozygote forms of the minor allele were merged to be compared with the homozygote of the major allele, and 3) the minor allele inheritance was considered recessive, and the homozygote forms of the minor allele were compared with merged remaining genotypes. In our data, the Hardy–Weinberg equilibrium principle was met. Therefore, we did not perform additional analysis.

Repeated measure analysis of variance (ANOVA) was used for comparison of genotype-based comparison of functional scores. *P* value (2-sided) > 0.05 was considered significant for all tests. All the statistical analyses were conducted utilizing the SPSS software version 22 (IBM, USA). Finally, the power of this study was calculated as 0.81 with GPower software.

## Results

Demographic and sports data are represented in Table 1. There was no significant difference between the ACL injured groups and the control group regarding age, BMI, type, and years of the sports. The distribution of the genotype was in accordance with the Hardy–Weinberg principle. Alleles (G or T) frequency in groups were summarized in Table 2. Compared with the ACL injury group, G allele frequency was non-significantly higher in the injured group with an odds ratio [95% CI] of 1.08 [0.79–1.47] (*P* = 64).

**Table 1:** Demographic and sport data

Variables (mean ± SD)	ACL injury (*n* = 200)	Control (*n* = 200)	*P* value
Age (years)	30.3 ± 6.4	29.7 ± 6.0	0.33
BMI (kg/m^2^)	24.1 ± 8.6	25.6 ± 9.1	0.09
Sports, *n* (%)			
Soccer	107 (53.5%)	110 (55%)	0.95
Volleyball	45 (22.5%)	46 (23%)	
Basketball	38 (19%)	36 (18%)	
Handball	10 (5%)	8 (4%)	
Years in sports	9.5 ± 5.9	9.2 ± 4.8	0.17

*ACL* anterior cruciate ligament, *BMI* Body Mass Index

**Table 2:** Allele distribution between case group and control group

Allele frequency	ACL injury (*n* = 200)	Control (*n* = 200)	OR [95% CI]	*P* value	HWE
Guanine (G)	291 (72.8%)	285 (71.2%)	1.08 [0.79–1.47]	0.64	1.0
Thymine (T)	109 (27.2%)	115 (28.8%)			1.0

*ACL* anterior cruciate ligament, *OR* odds ratio, *CI* confidence interval, *HWE* Hardy–Weinberg Equilibrium

Different genotype frequencies are shown in Table 3. Assuming all three possibilities for gene inheritance, including dominant, recessive, or codominant in a 2-by-3 general association test, we did not find a significant difference between cases and controls in genotype.

**Table 3:** Impact of *COL1A1* rs1107946 G/T polymorphism on ACL tear based on different genetic inheritance

Genetic inheritance	Genotype	ACL injury (*n* = 200)	Control (*n* = 200)	*P* value
Codominant, *n* (%)	GG	124 (62%)	114 (57%)	0.27
	GT	43 (21.5%)	57 (28.5%)	
	TT	33 (16.5%)	29 (14.5%)	
Dominant, *n* (%)	GG	124 (62%)	114 (52%)	0.31
	GT + TT	76 (38%)	86 (48%)	
Recessive, *n* (%)	TT	33 (16.5%)	29 (14.5%)	0.58
	GT + GG	167 (83.5%)	171 (85.5%)	

*ACL* anterior cruciate ligament, *OR* odds ratio, *CI* confidence interval, *G* Guanine, *T* Thymine, *GG/GT/TT* different single-nucleotide polymorphism of COL1A1 rs1107946

Clinical examinations (the ADT and Lachman test) and PROMs (IKDC score, KOOS score, Tegner Lysholm, and ACL-RSI) of the ACL tear group before and 1-year after surgery are represented in Table 4 in terms of genotypes. We witnessed significant improvement in these scores when comparing the pre-post values. However, none were significantly different between the three genotypes.

**Table 4:** Patients’ functional outcome pre- and 1-year postoperatively in terms of genotypes (mean ± SD)

Outcome	Preoperative			1-year postoperative			*P* value
	GG (*n* = 124)	GT (*n* = 43)	TT (*n* = 33)	GG (*n* = 120)	GT (*n* = 40)	TT (*n* = 30)	
Anterior knee pain (VAS)	5.0 ± 1.4	5.0 ± 1.6	4.9 ± 1.5	2.3 ± 1.1	2.6 ± 1.1	2.6 ± 1.1	Group effect: 0.80 Time effect: < 0.001 Interaction: 0.43
Drawer test (mm)	6.1 ± 1.2	6.3 ± 1.3	5.8 ± 1.4	1.5 ± 0.5	1.7 ± 0.7	1.5 ± 0. 6	Group effect: 0.09 Time effect: < 0.001 Interaction: 0.31
Lachman test (mm)	6.5 ± 1.2	6.3 ± 1.2	6.3 ± 1.2	1.5 ± 0.5	1.7 ± 0.6	1.6 ± 0.6	Group effect: 0.81 Time effect: < 0.001 Interaction: 0.16
IKDC knee score	63.5 ± 6.0	65.0 ± 8.0	61.4 ± 10.4	86.2 ± 4.1	86.1 ± 4.4	85.9 ± 3.5	Group effect: 0.27 Time effect: < 0.001 Interaction: 0.07
KOOS	61.7 ± 4.7	61.5 ± 4.9	60.3 ± 5.5	86.2 ± 4.1	86.1 ± 4.4	85.9 ± 3.5	Group effect: 0.50 Time effect: < 0.001 Interaction: 0.69
Tegner Lysholm	65.7 ± 4.4	65.0 ± 3.7	66.1 ± 4.2	88.4 ± 3.0	88.7 ± 2.6	89.0 ± 2.8	Group effect: 0.19 Time effect: < 0.001 Interaction: 0.53
ACL-RSI	29.1 ± 8.4	29.4 ± 7.8	27.8 ± 6.0	88.5 ± 7.8	88.9 ± 8.1	91.6 ± 8.8	Group effect: 0.73 Time effect: < 0.001 Interaction: 0.12

*Two-way repeated measure ANOVA

*VAS* visual analog scale, *IKDC* international knee documentation committee, *KOOS* knee injury and osteoarthritis outcome score, *ACL-RSI* ACL-Return to Sport after Injury, *G* Guanine, *T* Thymine

## Discussion

The current study aimed to evaluate whether the *COL1A1* rs1107946 (G/T) polymorphism is a risk factor for ACL injury in a population of elite sports players compared to a matched control group. Furthermore, we examined the discrepancy in response to ACL reconstruction surgery between patients with different genotypes regarding the *COL1A1* rs1107946 (G/T) polymorphism. Based on our findings, *COL1A1* gene polymorphism at rs1107946 (G/T) is not associated with a higher risk of ACL tear in young male professional athletes in the Middle East region. No significant difference was found in terms of genotype between the healthy and ACL groups in our study. In addition, responses to treatment and PROMs were not significantly different between genotype groups. The molecular and cellular pathways show that genetic variants might play a role in this kind of sports injury. However, the role of genetic variants in ACL tear is under debate, and our results are in line with other studies with a different population [18, 20, 23, 24].

Previous studies highlighted the importance of the genetic variants of the *COL1A1* gene in ACL pathologies [15, 18–20, 23–25]. In a systematic review by Kaynak et al., the association of 33 distinct DNA variants and ACL tear was analyzed [15]. These authors suggested no association of *COL1A1* rs1107946 and ACL injury, but with inadequate evidence. Stepien-Slodkowska et al. [18] studied 138 male Polish recreational skiers with primary ACL tear and did not report the genotype and allele frequency of rs1107946 as a risk factor for ACL tear incidence. Ficek et al. [23] investigated 91 Polish male professional soccer players with ACL tear and found COL1A1 rs1107946 and rs1800012 SNPs genotype distribution and allele frequency were not significantly different between the ACL tear group and controls. Also, they investigated the association between the COL1A1 rs1107946 and rs1800012 haplotypes and ACL tears. According to their study, G-T (rs1800012 and rs1107946) haplotypes may have a modest protective effect on ACL tears, when recessive inheritance patterns are assumed (*P* = 0.048). A cohort study by Sivertsen et al. [20] on 851 Scandinavian elite female athletes revealed no significant relationship between rs1107946 and the risk of ACL injury. Prabhakar et al. [24] researched 52 Indians with an ACL tear but otherwise normal knee and claimed the mentioned deduction. In conclusion, some of the previous studies do not support the role of rs1107946 SNP as a risk factor for ACL tear in professional athletes.

On the other hand, conflicts still prevail. A study by Stępień-Słodkowska [19] on 180 Polish male and female recreational skiers with primary ACL tear indicated a significant difference in the genotype of the injured ACL skiers (GG = 82.2%, GT = 16.7%, TT = 1.1%), in comparison with control subjects (GG = 71.4%, GT = 26.5%, TT = 2.2%). The GG genotype was predominant in the ACL injury group, compared to controls, and cases had a significantly higher G allele frequency in their study [19]. This study represents rs1107946 *COL1A1* as a potential factor projecting the ACL pathologies. However, the injury mechanism in ski is somehow different from other non-contact ACL injuries due to the specific nature of movements in this sport [18, 25].

The observed controversy in terms of *COL1A1* gene variants could be attributed to the complicated genetic background of ACL tears. We still do not have a clear viewpoint about this content due to insufficient evidence, a high risk of bias in the studies, and unclear conclusions. Therefore, further evaluations of genetic associations are recommended [15]. The *COL1A1* rs1107946 polymorphism is also studied in Achilles tendon rupture (ATR) [22]. A study conducted by Gibbon et al. analyzed banked DNA samples of white Europeans from South Africa and the United Kingdom and revealed that the G-T (rs1107946-rs1800012) haplotype was a preventive factor for acute Achilles tendon ruptures [22]. Their results are in line with those of Ficek et al. [23] about the ACL tear. A number of factors may have contributed to the controversial result of the role of COL1A1 rs1107946 polymorphism in the development of ACL injuries. First, several studies do not consider confounding factors such as the level of energy associated with trauma or the mechanism of injury. Second, the studies were conducted on different populations, including Norwegian and Finnish, Polish, South African, and UK athletes, as well as Iranian athletes for our study. Third, participants in previous studies were of varying sexes, some of them are males, some are females, and some both. Lastly, different recruitment of athletes from different disciplines and levels (elite, professional, or recreational) could also result in different association results.

Besides the knowledge of the underlying genetics of diseases, their application for early diagnosis, special treatment, and prognosis, or in other words, “Individualized Medicine,” should be highlighted. It has been proposed that the expression of SNPs in genes involved in the regeneration and repair of connective tissue may explain individual variations in injury severity, healing time, and injury rate [43, 44]. None of the previous studies in this context investigated patients' response to ACL reconstruction surgery in terms of *COL1A1* rs1107946 polymorphism. In the present study, one year after ACL reconstruction surgery, we observed that the functional knee scores were not different between distinct genotypes. Furthermore, physical examination for ACL stability was similar between diverse genotypes. Factors contributing to the response to treatment, such as age, gender, comorbidities, the duration and center of postoperative rehabilitation physical therapy, surgeon and surgical technique, as well as the graft site, were all the same in this study. As a result, we may conclude that the response of patients to treatment is not related to the background genetic variants of *COL1A1* rs1107946 (G/T). This result is being obtained from the Middle Eastern population for the first time and requires further investigation with more samples and comparison with other SNP in other parts of the world.

## Limitations

Our study encountered some limitations. We did not find a significant difference between genotypes for the outcomes of interest. However, we have to consider type 2 errors regarding the negative results. Nevertheless, the study sample size is appropriate compared with similar genetic studies [18–20, 23–25]. We could not figure out the exact causal relationship between genetic variants and the ACL tear incidence due to the case–control design of the study. Despite matching the cases and controls regarding age, ethnicity, BMI, years in the sport, and type of sport, heterogeneity among participants for other confounding factors is inevitable. Anatomical variations and other genetic factors concerning ACL tear (e.g., matrix metalloproteinase) may obscure the results [45, 46]. Finally, a longer follow-up may discover disparate results.

## Conclusion

According to the findings of this study, SNP at *COL1A1* rs1107946 (G/T) was not a predisposing genetic factor for ACL injury in a population of young professional male athletes in the Middle East. Furthermore, patients' responses to treatment were not different between distinct genotypes. Therefore, further investigations are necessary to determine the other possible genetic factors and protein production that lead to sport-related ligament injuries.

## Data Availability

The data that support the findings of this study are available from the corresponding author, H. Akbari Aghdam, upon reasonable request.

## Abbreviations

ACL: Anterior cruciate ligament
PROM: Patient-reported outcome measures
MRI: Magnetic resonance imaging
BMI: Body Mass Index
ADT: Anterior drawer tests
IKDC: International Knee Documentation Committee
KOOS: Knee Injury and Osteoarthritis Outcome Score
VAS: Visual Analog Scale
ACL-RSI: ACL-return to sport after injury
ADL: Activities of daily living
OR: Odds ratio
ANOVA: Analysis of variance
G: Guanine
T: Thymine
PCR: Polymerase chain reaction
